# Multi-Mode Integrated Bioinspired Electronic Tongue for Point-of-Care Tear Diagnosis

**DOI:** 10.3390/bios16080402

**Published:** 2026-07-24

**Authors:** Xiao-Xin Liang, Haochen Wu, Yong Wang

**Affiliations:** 1 Department of Ophthalmology, The Fourth Affiliated Hospital of School of Medicine, International School of Medicine, International Institutes of Medicine, Zhejiang University, Yiwu 322000, China; 8016046@zju.edu.cn; 2 Department of Mechanical Engineering, Hangzhou City University, Hangzhou 310015, China; 2250306022@stu.hzcu.edu.cn

**Keywords:** triboelectric nanogenerator, tear analysis, bioinspired sensing, microfluidic chip, electronic tongue

## Abstract

Tear analysis plays a crucial role in the early screening and diagnosis of ophthalmic diseases. However, conventional methods are often limited by poor real-time performance, low portability, and insufficient capability for multi-parameter detection. Here, we present a bioinspired triboelectric electronic tongue integrated with a microfluidic chip for multimodal detection of tear pH and disease-related biomarkers. The system combines three triboelectric nanogenerator (TENG) modes, including droplet-based, dual-electrode sliding, and single-electrode sliding configurations. The droplet-based TENG converts gravitational potential energy into electrical energy, generating a maximum output voltage of 65 V. The sliding TENG further expands the sensing dimensions by characterizing droplet flow behavior and viscosity-related properties. Benefiting from the high sensitivity of the dual-electrode mode and the waveform differentiation capability of the single-electrode mode, the platform enables enhanced sample discrimination. After optimizing key parameters, including droplet height and chip inclination angle, the output stability errors for all three TENG modes were maintained within ±10%. Combined with a random forest algorithm, the multimodal sensing system achieved a classification accuracy exceeding 96.6% for artificial tears with different pH values. Moreover, distinct electrical response patterns were observed for ophthalmic disease-related biomarkers, including Lysozyme, Interleukin-6 (IL-6), and Chlamydia, demonstrating excellent type identification and concentration detection capability. This work provides a self-powered and miniaturized strategy for intelligent tear analysis and multiple-parameter sensing, offering significant potential for ophthalmic disease diagnosis.

## 1. Introduction

Human body fluids play essential roles in metabolic regulation and contain a wide range of biomarkers, including electrolytes, proteins, nucleic acids, and metabolites, which can reflect the physiological and pathological states of the body [1,2,3]. Accordingly, body fluid analysis has become an important tool for early disease screening, therapeutic evaluation, and personalized healthcare [4,5]. Among various body fluids, tears are particularly attractive for diagnostic applications because they can be collected in a relatively convenient and minimally invasive manner. The physicochemical and biochemical characteristics of tears, such as pH, ionic composition, and lysozyme level, are closely associated with ocular surface health [6,7]. Previous studies have shown that decreased tear lysozyme levels are commonly related to dry eye-associated inflammatory responses, reduced antimicrobial protection, and disruption of the ocular surface microenvironment. Meanwhile, abnormal tear pH variations, especially alkaline shifts, may be associated with bacterial keratitis, chemical injury, or impaired ocular surface homeostasis [8,9]. Therefore, tear analysis holds considerable potential for supporting the diagnosis and disease assessment of dry eye disease, infectious ocular disorders, and autoimmune-related ocular diseases [10,11,12].

Despite this potential, existing tear diagnostic methods still face several limitations in practical applications. Laboratory-based methods, including pH meters, electrolyte analyzers, mass spectrometry, and lysozyme assay kits, generally provide relatively high analytical accuracy. However, they often require sophisticated instruments, sample pretreatment, and professional operation, which limits their use in rapid on-site testing and point-of-care diagnosis [13,14,15,16]. For example, electrolyte analyzers usually involve sample pretreatment and electrode-based measurements, resulting in relatively cumbersome procedures and increased detection costs. In contrast, portable approaches, such as pH test strips and commercial dry eye diagnostic systems, are easier to operate but are usually limited to single-parameter or limited-parameter analysis. For instance, pH test strips typically provide a resolution of approximately 0.5 pH units, which may be insufficient to identify subtle pH variations of 0.2–0.3 units in patients with dry eye disease. Most commercial dry eye diagnostic systems mainly evaluate tear film break-up time and tear secretion volume, such as the Schirmer test, but cannot simultaneously provide multidimensional biochemical information, including tear pH, ionic composition, protein levels, and inflammatory biomarkers [17,18]. In addition, tear samples are typically available only at microliter volumes, and their composition can be dynamically affected by blinking, tear film distribution, tear secretion status, and inter-individual variability, which further increases the difficulty of accurate detection and comprehensive analysis [19]. Therefore, there is an urgent need to develop a miniaturized, intelligent, and multimodal tear analysis platform capable of directly analyzing microliter-scale tear samples while enabling multiparameter sensing, self-powered operation, portable testing, and potential applications in home healthcare and telemedicine.

The electronic tongue (E-tongue) is a liquid sensing system based on sensor arrays and pattern recognition algorithms, inspired by gustatory neurophysiology [20,21,22]. It generates characteristic response signals through interactions between sensor elements and target analytes, enabling data-driven interpretation of liquid composition. For example, electrochemical sensors detect variations in ion concentration by converting them into potential signals, while optical sensors transduce protein-related information via fluorescence intensity changes [23]. Although the E-tongue has achieved significant progress in taste analysis and chemical sensing, its application in outdoor and home-based health monitoring remains limited due to the insufficient versatility of sensing mechanisms and reliance on conventional battery-powered operation [24]. Such power-dependent systems are often constrained by limited operational lifetime, high maintenance cost, and environmental concerns, which hinder long-term deployment and large-scale adoption [25]. Therefore, the development of a self-powered, structurally simple, and cost-effective droplet-based electronic tongue has become an important research direction in this field.

Recently, triboelectric nanogenerators (TENGs) have attracted considerable attention due to their efficient mechanical energy harvesting capabilities [26,27,28,29,30]. The triboelectric signals generated during operation have also been extensively explored for liquid identification, enabling the extraction of an “electrical fingerprint” through contact electrification between droplets and solid surfaces for compositional analysis and classification [31,32]. The triboelectric electronic tongue combines triboelectric effects with multichannel sensing strategies. Its working principle relies on the generation of characteristic electrical signals when different liquids interact with an identical solid surface, arising from variations in physicochemical properties such as polarity, surface tension, and conductivity. By engineering controllable triboelectric responses via contact-separation processes and leveraging machine learning algorithms for feature extraction and classification, high-precision liquid identification can be achieved [33,34]. However, conventional liquid–solid TENGs are limited by relatively low energy conversion efficiency, stemming from two-dimensional interfacial charge-transfer mechanisms that yield weak output signals and reduced sensing sensitivity [35]. Droplet electricity generators (DEGs) partially address this issue by extending the charge transfer pathway from a two-dimensional interface to the three-dimensional droplet volume, thereby significantly enhancing signal amplitude. Nevertheless, their inherently single-peak waveform limits the ability to differentiate between complex liquid samples [36]. Therefore, integrating multiple TENG operating modes (e.g., contact-separation and sliding modes) onto a single microfluidic chip, combined with artificial intelligence-based multidimensional signal analysis, offers a promising strategy to overcome the limitations of single-mode systems and significantly improve the accuracy and practical applicability of tear analysis [37].

In this work, we propose a multi-mode integrated bioinspired electronic tongue (MIBET) for online droplet-based tear analysis. The system integrates three distinct TENG mechanisms, drop-based mode (Section S1), dual-electrode sliding mode (Section S2), and single-electrode sliding mode (Section S3), into a single microfluidic chip, enabling simultaneous detection of tear pH and disease-related biomarker concentrations. The drop-based TENG converts gravitational potential and kinetic energy arising from the height difference between droplets and polymer films into high-amplitude electrical signals. The sliding TENG further deciphers droplet flow dynamics and viscosity-related properties, where the dual-electrode mode provides high output and sensitivity, while the single-electrode mode enhances sensing dimensionality through waveform differentiation. The output stability of different modes was systematically evaluated based on charge-accumulation effects, revealing distinct performance variations among configurations. To optimize device performance, the effects of droplet release height, device tilt angle, and droplet dripping velocity on signal output were comprehensively investigated, and optimal operating conditions were identified. Finally, the multimodal sensing platform was coupled with a random forest algorithm for the analysis of artificial tear samples with varying pH values. The results demonstrate that all TENG modes achieve high classification accuracy (≥96.6%). Moreover, the system exhibits distinct electrical response patterns toward artificial tears individually doped with different disease biomarkers, confirming its capability for multiplexed biomarker detection. Overall, this work advances tear analysis toward miniaturization, intelligence, and personalization, providing a promising strategy for next-generation self-powered smart diagnostic systems.

## 2. Materials and Methods

### 2.1. Conceptual Design of the Bioinspired Electronic Tongue for Tear Analysis

**Figure 1:**
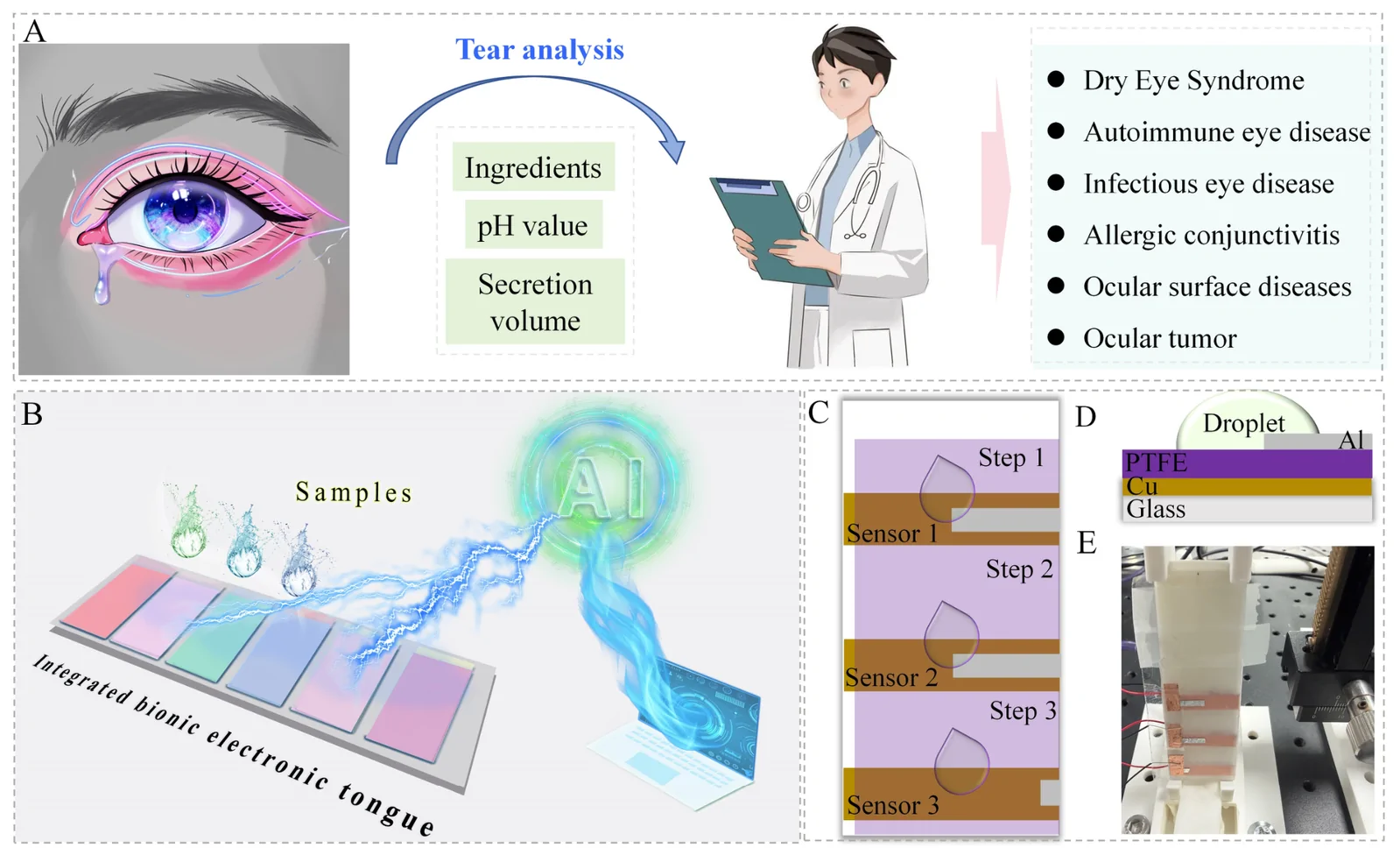
Working principle and structural design of MIBET. (**A**) Schematic illustration of ophthalmic disease diagnosis via compositional analysis of a single tear sample. (**B**) Conceptual design of an intelligent biomimetic electronic tongue. (**C**) Structural diagram of the multimodal integrated droplet sensor. (**D**) Side-view schematic of a dual-electrode droplet generator operating in dual-electrode mode. (**E**) Photograph of the fabricated MIBET device.

As illustrated in Figure 1A, variations in tear composition are closely associated with ocular health status [38,39,40]. Accordingly, this study proposes a bioinspired electronic tongue-based tear analysis system, as shown in Figure 1B. Inspired by biological gustatory sensing mechanisms, the system integrates multimodal triboelectric sensing technologies to enable high-precision, real-time, and non-invasive tear analysis [41]. Built on a microfluidic chip platform, multiple triboelectric operating modes are monolithically integrated into a single device, mimicking the multidimensional sensing capability of biological taste buds toward liquid physicochemical properties. Through large-scale stable measurements combined with machine learning algorithms, the system enables simultaneous decoding of tear pH, lysozyme activity, and disease biomarker signatures, thereby establishing an “electrochemical fingerprint database”. This conceptual design not only overcomes the limitations of conventional diagnostic methods but also endows the system with self-powered, miniaturized, and intelligent features. It provides a promising pathway for home-based self-testing, telemedicine, and personalized diagnostics, and is expected to facilitate the transition of tear analysis from laboratory to POCT applications [42].

### 2.2. Fabrication of the Bioinspired Triboelectric Electronic Tongue

As shown in Figure 1C, the MIBET device integrates three distinct triboelectric droplet sensors within a unified material system. The layered structure, from bottom to top, consists of a glass substrate, a copper electrode, a polytetrafluoroethylene (PTFE) film, and an aluminum electrode, meeting the requirements for low-cost device fabrication. Specifically, the glass substrate measures 75 × 25 mm, while a dual-sided conductive copper tape (0.5 × 30 mm) is attached to the backside of the PTFE film (50 × 25 mm). A portion of the copper tape is extended to the rear side of the glass substrate to enable electrical interconnection. The entire assembly is encapsulated on the front side of the glass substrate using double-sided adhesive tape. For the dual-electrode droplet generator, an aluminum electrode (0.2 × 25 mm) is directly attached to the front surface of the PTFE film and precisely aligned with the underlying copper electrode. In contrast, the single-electrode droplet generator employs a shortened aluminum electrode that avoids direct contact with liquid droplets, serving primarily as a wiring terminal to minimize signal noise. As illustrated in Figure 1D, the dual-electrode configuration incorporates an additional top aluminum electrode, which distinguishes its structural design from the single-electrode mode.

The fabricated triboelectric bioinspired electronic tongue, shown in Figure 1E, clearly demonstrates the complete device architecture. The material selection is guided by functional considerations: the PTFE film acts as the solid triboelectric layer, leveraging its high electron affinity and intrinsic superhydrophobicity to suppress unwanted solid–liquid interfacial charge dissipation, thereby ensuring efficient energy and charge transfer [43]. The liquid droplet serves as the liquid triboelectric counterpart. Copper foil is selected as the electrode material due to its excellent electrical conductivity, low cost, and commercial availability. Both PTFE and copper foil are prepared to industrial-grade specifications, providing high surface flatness, mechanical durability, and stable energy conversion performance. Experimental results confirm that the device maintains stable operation over extended use after a single activation. Conductive wires with a diameter of 0.6 mm are used to connect the copper electrodes, enabling accurate acquisition and measurement of triboelectric signals [44].

### 2.3. Preparation of Liquid Samples and Construction of the Experimental Platform

To systematically evaluate the fundamental performance of the MIBET, deionized water was used as the control sample for baseline measurements, while artificial tears (purchased from Yangtuo Automation Technology Co., Ltd., Dongguan, China) were employed as simulated physiological samples for characterization experiments. Gradient artificial tear samples with varying pH values (pH 6.0–9.0) were custom-prepared by the supplier, covering the pH range typically observed in tear fluids under common pathological conditions. In addition, biomarker samples (e.g., lysozyme and inflammatory factors) were obtained from Yeasen Biotechnology Co., Ltd. (Shanghai, China) and diluted to a specific concentration gradient to validate the system’s capability for disease-related biomarker detection. For each pH group, artificial tear samples were independently prepared or aliquoted before measurement. Each independent solution batch was measured separately, and waveform segments obtained from the same measurement run were assigned to the same data group.

The MIBET device was mounted on a 3D-printed holder at different inclination angles (15°, 30°, 45°, and 60°). The holder was designed to ensure both mechanical stability and adjustable orientation, thereby guaranteeing high experimental repeatability. Liquid droplets were delivered using portable droppers (inner diameter: 0.8 mm, flow accuracy: ±5%), and the vertical distance between the droplet outlet and the sensor surface was adjusted within a range of 2–20 mm. A three-degree-of-freedom graduated translation stage (positioning accuracy of X/Y/Z axes: 0.1 mm) was used to precisely control the spatial position of the dropper, ensuring that droplets impacted the sensor surface at defined angles (vertical or inclined) while minimizing mechanical disturbances during signal acquisition. The six electrode leads were connected to an oscilloscope (RTB2004, Rohde & Schwarz, Munich, Germany) via high-voltage probes (impedance: 50 Ω, bandwidth: 100 MHz), forming a real-time three-channel triboelectric sensing array. The preprocessed data were then used to construct classification models based on the Random Forest (RF) algorithm, implemented using the scikit-learn library in Python 3.14.6.

### 2.4. Comparison of Working Mechanisms Between Single-Electrode and Dual-Electrode DEG Modes

**Figure 2:**
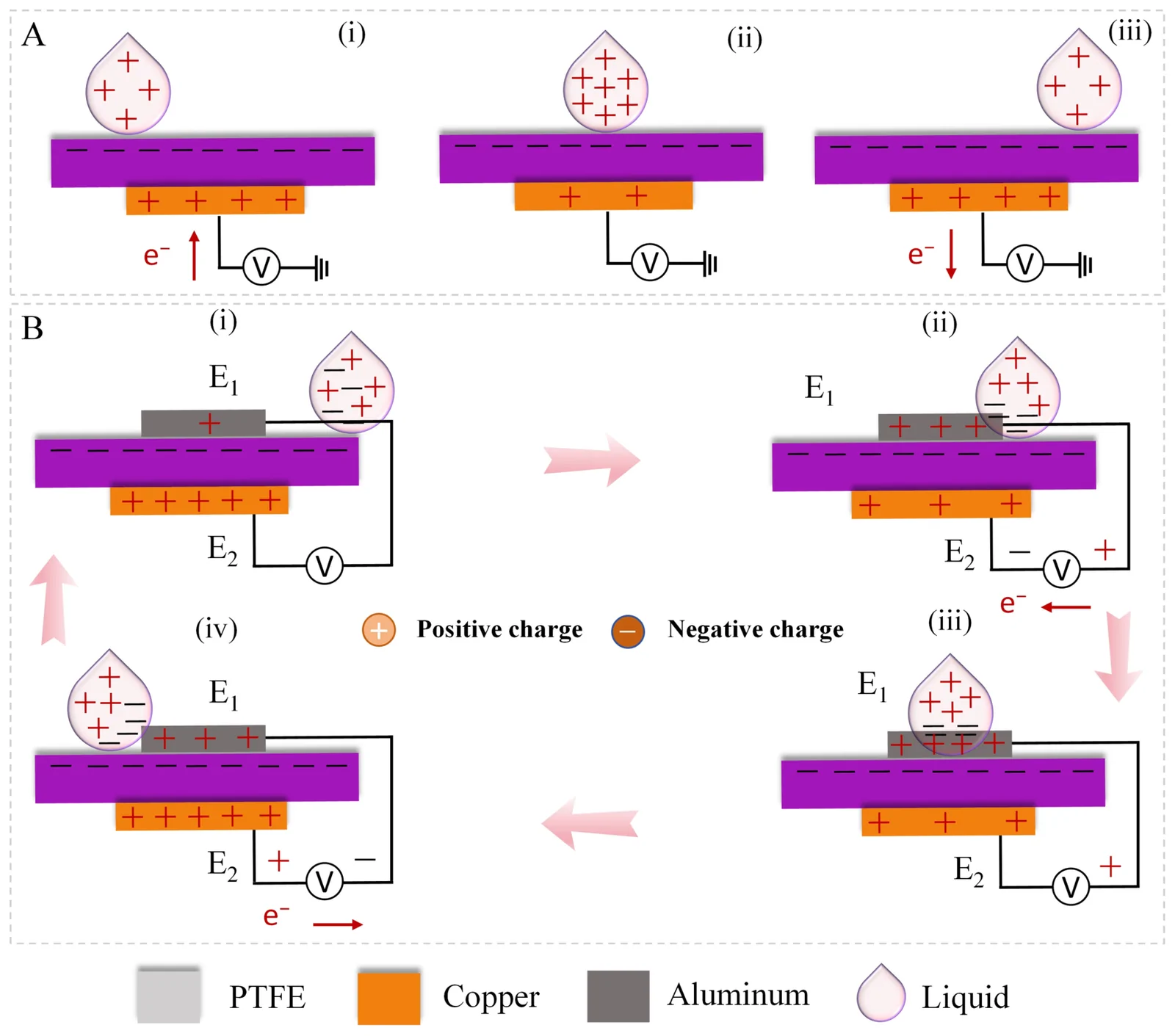
Working mechanism of the biomimetic triboelectric electronic tongue. Dynamic charge behaviors in (**A**) the single-electrode mode and (**B**) the dual-electrode DEG mode. The red arrows denote the direction of electron flow.

Figure 2 illustrates the dynamic charge behaviors in the single-electrode mode and the dual-electrode DEG mode. As shown in Figure 2A, when droplets slide across the polytetrafluoroethylene (PTFE) film, contact electrification occurs at the interface due to the difference in electronegativity between water droplets and PTFE. Electrons are transferred from the water droplets to the PTFE film, resulting in positively charged droplets and negatively charged PTFE, while the overall system remains electrically neutral. With repeated droplet–PTFE interactions, the surface charge density on the PTFE film and copper electrode gradually approaches saturation. When a droplet approaches the copper electrode, an electric double layer (EDL) forms at the PTFE–droplet interface, partially screening the negative charges on the PTFE surface. To balance the potential difference between the dielectric layer and the electrode, electrons flow from the ground to the copper electrode (State (i)). When the droplet fully contacts the copper electrode, the positive charges carried by the droplet neutralize the negative charges on the PTFE surface (State (ii)). As the droplet detaches from the copper electrode, electrons flow back from the copper electrode to the ground (State (iii)). This process repeats cyclically until the droplet completely leaves the surface and the system returns to electrostatic equilibrium. Periodic electrical output signals can be obtained by monitoring the potential difference between the ground terminal and the copper electrode [45].

The dynamic charge behavior under the dual-electrode mode is illustrated in Figure 2B. PTFE possesses excellent negative charge storage capability. Through electrostatic induction between the inner and outer electrodes, electric fields E1 and E2 are established. When the droplet is not in contact with the inner electrode, the system can be modeled as two series-connected capacitors, *C_m_* and *C_n_* (State (i)). Here, *C_m_* is formed at the inner electrode-air-water interface, while *C_n_* represents the capacitance of the PTFE dielectric layer. Although charge redistribution occurs between the liquid and PTFE, no measurable open-circuit voltage is generated in this state. When the droplet contacts the inner electrode, *C_m_* is replaced by *C*_1_ and *C*_2_ (State (ii)). In this case, *C*_1_ corresponds to the electric double-layer (EDL) capacitance at the liquid-PTFE interface, and *C*_2_ represents the EDL capacitance at the liquid-inner electrode interface. Under the combined influence of *E*_1_ and *E*_2_, negative charges within the droplet migrate toward the inner electrode, while positive charges accumulate near the PTFE interface. This disrupts the potential equilibrium between the inner and outer electrodes and drives positive charges on the outer electrode to flow toward the inner electrode, thereby generating an output voltage (State (iii)). As the droplet moves away from the inner electrode region (State (iv)), the electric double layer at the water-PTFE interface gradually dissipates, while the induced charges on the PTFE surface drive positive charges on the inner electrode back toward the outer electrode. Once the droplet fully departs from the inner electrode, the electrode potential returns to its initial state. This fully reversible process establishes a closed-loop kinetic model for charge transfer at the liquid–solid interface [46].

Overall, the three sensing modes of the MIBET system are primarily governed by liquid–solid triboelectrification, electric-double-layer modulation, and electrostatic induction. Importantly, these modes do not represent independent sensing mechanisms; rather, they serve as complementary implementations of liquid–solid triboelectric sensing under different droplet motion behaviors and electrode configurations. Specifically, the droplet mode generates high-amplitude signals associated with droplet impact, contact–separation processes, and interfacial charge accumulation. The dual-electrode sliding mode enhances the system’s response to charge transfer, electrical conductivity, ionic strength, and electric-double-layer modulation. In contrast, the single-electrode sliding mode provides waveform features related to sliding dynamics, wettability, and interfacial charge evolution. By integrating these three sensing modes, the MIBET platform can extract multidimensional electrical fingerprints of tear samples from signal amplitude, waveform profile, and dynamic response behavior [47,48], thereby improving the reliability of tear pH recognition and disease-related biomarker analysis.

## 3. Results and Discussion

### 3.1. Output Characteristics and Stability of Different TENG Modes

**Figure 3:**
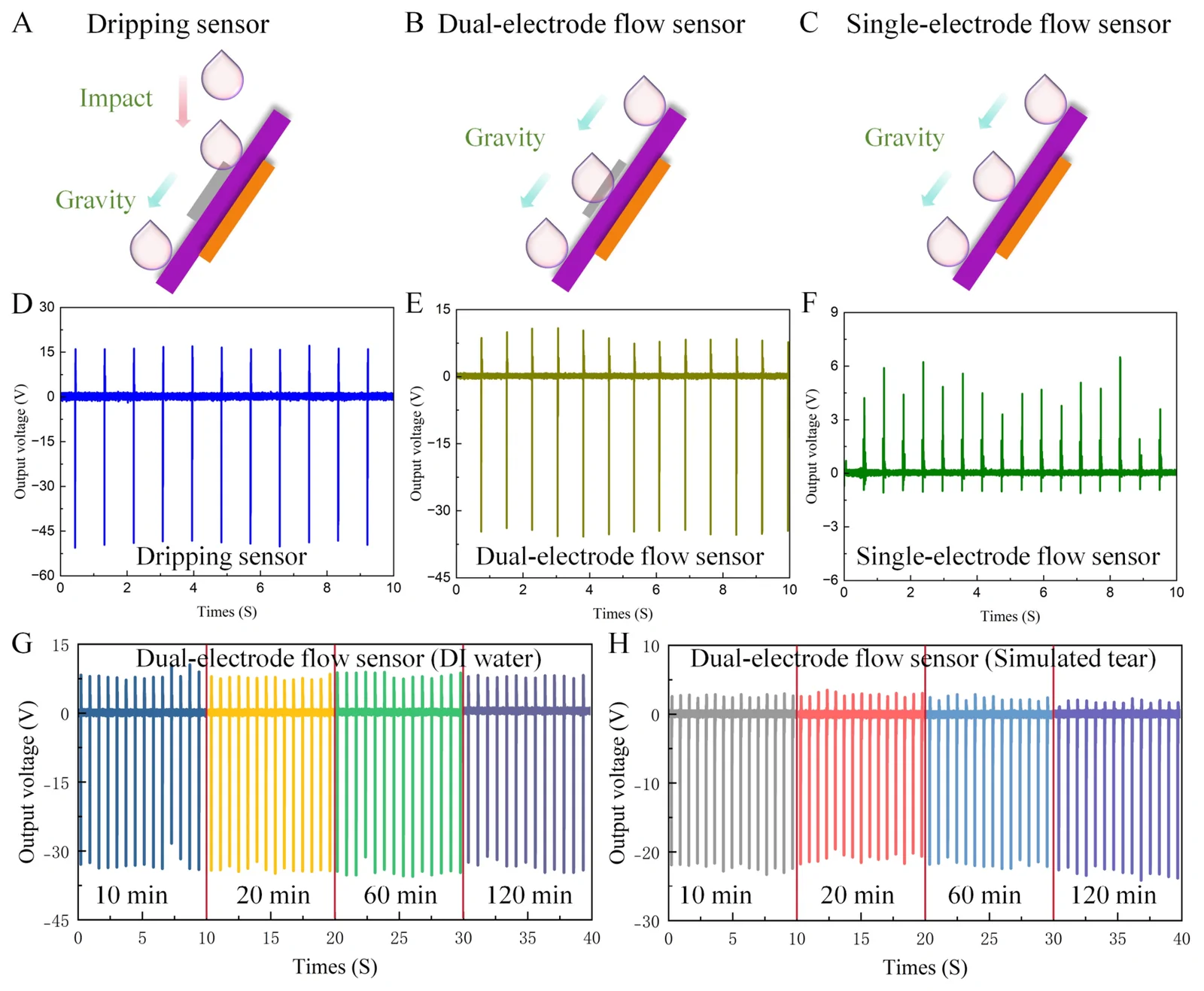
Basic output characteristics and stability analysis of a biomimetic triboelectric electronic tongue. (**A**–**C**) Schematic illustrations showing the theoretical output differences among the three TENG modes. (**D**–**F**) Measured open-circuit voltages of the three TENG modes (experimental conditions: height difference of 8 cm, inclination angle of 30°, droplet frequency of 75 drops per minute). Stability tests of the dual-electrode sliding-mode TENG, demonstrating stable output signals for both (**G**) deionized water and (**H**) artificial tears during 120 min of continuous operation.

Based on the principle of liquid–solid triboelectrification, the electrical output characteristics of the MIBET device are similar to those of conventional triboelectric nanogenerators (TENGs), exhibiting typical features of high voltage, low current, and high impedance [34]. Figure 3A–C show schematic diagrams of the three TENG operating modes. For the droplet-based TENG mode, the sensing signal is mainly generated during the impact, contact, spreading/retraction, and separation of falling droplets on the PTFE surface [35,49]. During this process, the gravitational potential energy and impact kinetic energy of the droplet are converted into electrical signals through liquid–solid contact electrification and electrostatic induction. Therefore, this mode generally produces the highest voltage output among the three modes. Its signal characteristics are mainly affected by droplet impact dynamics, effective contact area, contact duration, spreading behavior, surface tension, wettability, and charge-screening effects associated with electrical conductivity and ionic strength at the liquid–PTFE interface [50,51]. For the dual-electrode sliding-mode TENG, the output signal originates from the coupled effects of liquid–solid triboelectrification, electric-double-layer modulation, and charge redistribution between the two electrodes during droplet sliding [52,53]. Because the dual-electrode configuration provides an enhanced potential difference and a well-defined charge-transfer pathway, this mode is particularly sensitive to interfacial charge transfer, electrical conductivity, ion concentration, ionic strength, and electric-double-layer capacitance. Accordingly, it generally exhibits a relatively high voltage response and good sensitivity to variations in the physicochemical properties of tear samples. In contrast, the signal from the single-electrode sliding-mode TENG mainly arises from electrostatic induction between the charged droplet/PTFE interface and a single electrode connected to the external circuit [49,53]. Although the output amplitude of this mode is usually lower than that of the droplet-based and dual-electrode modes, its waveform is highly sensitive to droplet sliding velocity, contact-line pinning, wettability, viscous resistance, contact-area evolution, and interfacial charge accumulation/release processes. Therefore, this mode provides abundant dynamic waveform information related to droplet motion and interfacial interactions.

In the experiments, deionized water was loaded into a reservoir connected to a syringe-based injection system, and the open-circuit voltages of the three sensing modes were measured under fixed conditions: a height difference of 8 cm, an inclination angle of 30°, and a droplet frequency of 75 drops/min. Figure 3D–F show the output voltage signals of the three TENG-mode sensors under these testing conditions. For comparison, only the negative-polarity output signals are presented; unless otherwise specified, all comparative results in this study refer to negative output signals. As shown, the peak voltage reached 50 V for the droplet-based mode and 35 V for the dual-electrode sliding mode, whereas the single-electrode sliding mode generated only 4–5 V. These experimental results are in good agreement with the theoretical analysis.

Stable output is a critical performance metric for sensors in clinical applications. Using the dual-electrode sliding-mode TENG, continuous signal acquisition was conducted over 120 min with deionized water and neutral artificial tears as test samples. Figure 3G,H present representative segments of the recorded signals. The device maintained stable output with fluctuations within ±10% throughout long-term operation, confirming its excellent reliability. Notably, the output amplitude obtained with artificial tears (20–25 V) was significantly lower than that of deionized water (35 V). This trend is consistent with previously reported effects of electrolyte ion concentration on triboelectric output [34], further validating the system’s detection sensitivity and its applicability in practical sensing scenarios.

### 3.2. Structural Parameter Analysis and Output Optimization

**Figure 4:**
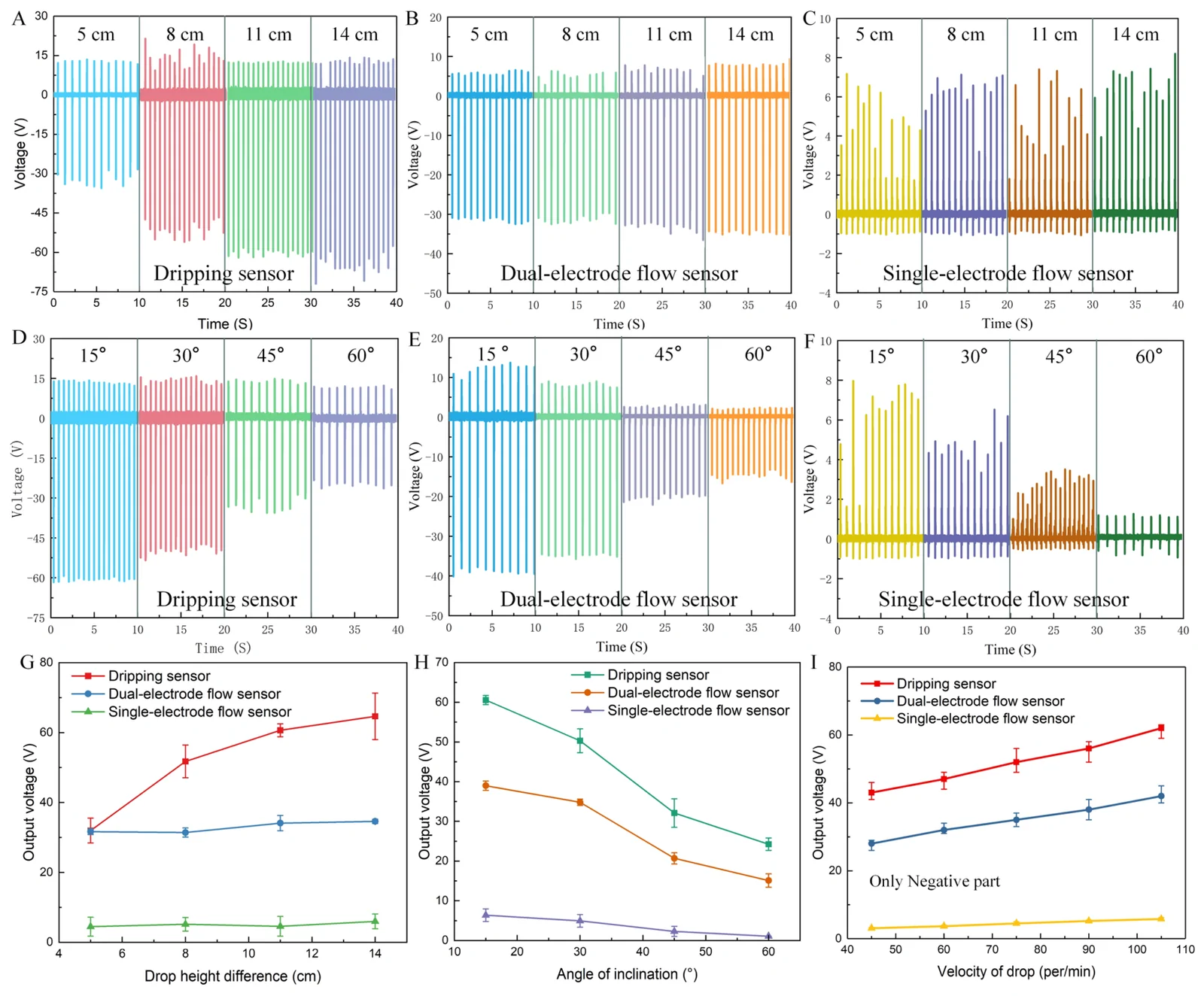
Structural parameter analysis and output optimization. (**A**–**C**,**G**) Effects of droplet falling height on the output voltages of the three TENG sensors with different working modes. (**D**–**F**,**H**) Effects of inclination angle on the output voltages of the three TENG sensors with different working modes. (**I**) Effects of dripping rate on the output voltages of the three TENG sensors with different working modes.

The performance of the MIBET is influenced by multiple structural parameters, including droplet falling height, inclination angle, and dripping rate. Systematic characterization was therefore conducted to optimize sensor performance. As shown in Figure 4A–C,G, the effect of droplet falling height on the output amplitude of the MIBET was first investigated. Using a three-axis translation stage, the falling height was precisely adjusted from 5 cm to 14 cm, and the output voltages of the three sensing modes were recorded simultaneously. The results show that the negative-polarity output voltage of the droplet-mode TENG increases significantly with increasing falling height, rising from 30 to 35 V at 5 cm to approximately 65 V at 14 cm. This enhancement is primarily attributed to the increased gravitational potential energy. In contrast, the negative-polarity outputs of the two sliding-mode sensors (dual-electrode and single-electrode TENGs) exhibit negligible variation, remaining at approximately 30–35 V and 5–7 V, respectively, since the sliding-mode devices do not rely on gravitational potential energy conversion.

The inclination angle determines the flow pathway of droplets between sensing units and facilitates waste-liquid collection. Therefore, its effect on signal output was also evaluated. Figure 4D–F,H present the output voltages of the three sensing modes at four different inclination angles. Overall, the output voltage decreases as the inclination angle increases. Here, the inclination angle is defined as the angle between the glass substrate and the normal direction of the vertical. Specifically, the droplet-mode TENG reaches a maximum negative-polarity output of 60–65 V at an inclination angle of 15°, corresponding to optimal sliding dynamics. When the angle increases to 60°, the output decreases to approximately 25 V. Similarly, the dual-electrode sliding-mode TENG output decreases from 40 V at 15° to 15 V at 60°, while the single-electrode mode decreases from 6 to 7 V to approximately 1 V. These results indicate that even a small angular deviation (±5°) can induce output fluctuations exceeding 20%. Therefore, a fixed-angle support structure is essential in practical applications to minimize recalibration and to improve measurement accuracy and long-term stability.

Finally, the influence of the dripping rate on the negative-polarity output was investigated by adjusting the flow-control valve on the infusion tube. The results demonstrate that the output voltage of all three sensing modes increases markedly with increasing dripping rate. As shown in Figure 4I, the droplet-mode TENG output rises from 40 to 45 V at 45 drops per minute to 60–65 V at 105 drops per minute. For the dual-electrode sliding-mode TENG, the output increases from 30 to 37 V to 40–45 V, while the single-electrode mode increases from 3 to 3.5 V to 5.5–6 V. This trend confirms that enhanced droplet kinetic energy directly improves device output performance, providing a theoretical basis for dynamic optimization of operational parameters.

### 3.3. Intelligent Sensing of Artificial Tears with Different pH Values

**Figure 5:**
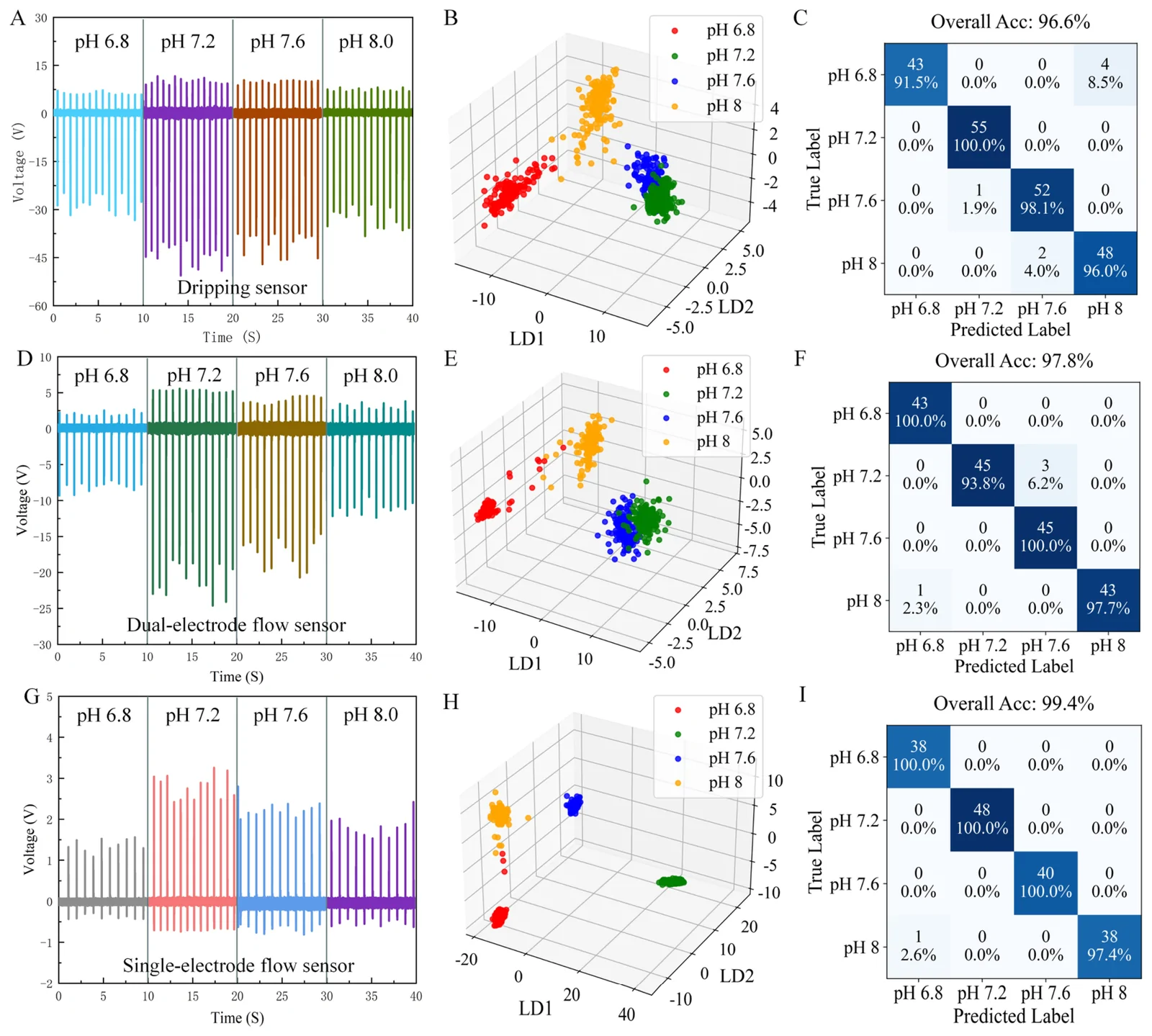
Intelligent sensing of artificial tears with different pH values. (**A**,**D**,**G**) Output voltage signals of three TENG sensors in artificial tears across four pH levels (6.8/7.2/7.6/8.0). (**B**,**E**,**H**) Visualization of feature dimensionality reduction for artificial tears with different pH values using the LDA algorithm, corresponding to the three TENG sensors. (**C**,**F**,**I**) Confusion matrices of classification results based on the Random Forest algorithm for the three TENG sensors. The classification accuracies for tear pH detection using the three TENG sensors are 96.6%, 97.8%, and 99.4%, respectively.

To validate the pH sensing capability of the MIBET for artificial tears, systematic experiments were conducted on four groups of artificial tears with graded pH values (6.8/7.2/7.6/8.0) using TENG sensors operating in three modes. The experimental results reveal a strong correlation between pH variation and the output signals across all sensing modes. As the pH shifts toward either acidic or alkaline conditions, the peak negative output voltage generally decreases, with more pronounced variations observed under stronger acidic conditions. Specifically, the droplet-mode TENG exhibits a nonlinear response, in which the negative peak output voltage first increases and then decreases over the pH range of 6.8 to 8.0 (30 V → 45 V → 40 V → 35 V), reaching its maximum at a mildly alkaline pH of 7.2, as shown in Figure 5A. The dual-electrode sliding-mode TENG demonstrates enhanced sensitivity to pH variations, exhibiting a clear gradient in output voltage with changing pH (10 V → 25 V → 20 V → 15 V), as shown in Figure 5D. Notably, the voltage difference between the acidic condition (pH 6.8) and the neutral condition (pH 7.2) reaches 15 V. Although the single-electrode sliding mode exhibits a relatively low output amplitude (1.5–3 V), its output voltage decreases monotonically with increasing pH, as shown in Figure 5G. In particular, the voltage approximately doubles as pH increases from 6.8 to 7.2, indicating a favorable linear response to pH variations. Overall, these results demonstrate that all three TENG modes are capable of effectively distinguishing artificial tears with different pH values. Among them, the dual-electrode sliding-mode sensor provides the highest sensitivity for pH gradient detection, while the single-electrode sliding-mode sensor exhibits superior linear response characteristics.

To overcome the limitations of manual liquid-sample classification and visual interpretation of complex waveform signals, machine-learning-based analysis was introduced to process the output signals of the MIBET system. All experiments were conducted under controlled environmental and operational conditions, and a standardized data processing pipeline was established to improve the reliability and reproducibility of the results. Considering the nonlinear and multifactorial characteristics of TENG signals, as well as the requirement for noise robustness under small-sample conditions, Random Forest was selected as the final classification model. Its ensemble learning framework is well-suited for capturing complex nonlinear relationships, and its robustness to noise and outliers contributes to stable classification performance. In addition, Random Forest can provide feature-importance rankings, which help identify important waveform regions or waveform-derived features associated with pH discrimination. LDA was used for supervised dimensionality reduction and class separability assessment rather than as the final classifier. By leveraging pH labels, LDA maximizes inter-class variance and minimizes intra-class variance, enabling intuitive visualization of the discrimination among different pH groups. The detailed data processing procedure was as follows. Continuous triboelectric voltage signals recorded by the oscilloscope were first segmented into single-cycle waveforms through peak detection. The segmented waveforms were then resampled to a fixed length using linear interpolation, denoised by low-pass filtering, and downsampled for data compression. The resulting one-dimensional voltage sequences were normalized and used as input features. To avoid data leakage, waveform segments derived from the same continuous recording, solution batch, or device were not simultaneously assigned to both the training and testing sets. This group-wise splitting strategy ensured that highly correlated waveform segments were not shared across the training and testing subsets, while satisfying the uniform dimensionality requirement for machine-learning inputs. This preprocessing strategy generated 125–182 analyzable segmented waveform samples, as listed in Section S1 of the Supporting Information. The dataset was then split into training and testing sets at a ratio of 7:3, with 70% of the data used for model training and 30% for testing. LDA was applied to evaluate class separability, as shown in Figure 5B,E,H, and the normalized features were subsequently imported into the Random Forest model for quantitative classification. The results show that artificial tear samples with four distinct pH values form well-separated clusters in the LDA feature space. A classification model was further constructed based on the Random Forest algorithm, and its performance was evaluated using confusion matrices, where the diagonal elements directly reflect agreement between predicted and true labels. The classification accuracies of the three TENG modes reached 96.6%, 97.8%, and 99.4%, respectively, as shown in Figure 5C,F,I. These results demonstrate the good classification performance of the model under the current validation protocol and highlight the strong discriminative capability of the sensor output signals. The consistency between the classification results and the LDA visualization further supports the effectiveness of the MIBET system in distinguishing artificial tears with different pH values.

In addition, as detailed in Section S2 of the Supporting Information, 5-fold and 10-fold stratified cross-validation were conducted as additional evaluations of model performance. The results further confirmed the robustness of the classification model. Therefore, this multimodal sensing strategy provides a solid technical foundation for real-time tear pH monitoring and is expected to facilitate the development of TENG-based diagnostic technologies for the ocular surface microenvironment. To further evaluate the scientific validity, algorithmic suitability, and adaptability of the proposed approach, we compared our method with representative tear-analysis methods and electronic-tongue-based sensing strategies reported in previous studies. The detailed comparison is summarized and discussed in Section S3 of the Supporting Information.

### 3.4. Detection and Analysis of Artificial Tears Individually Spiked with Different Biomarkers

**Figure 6:**
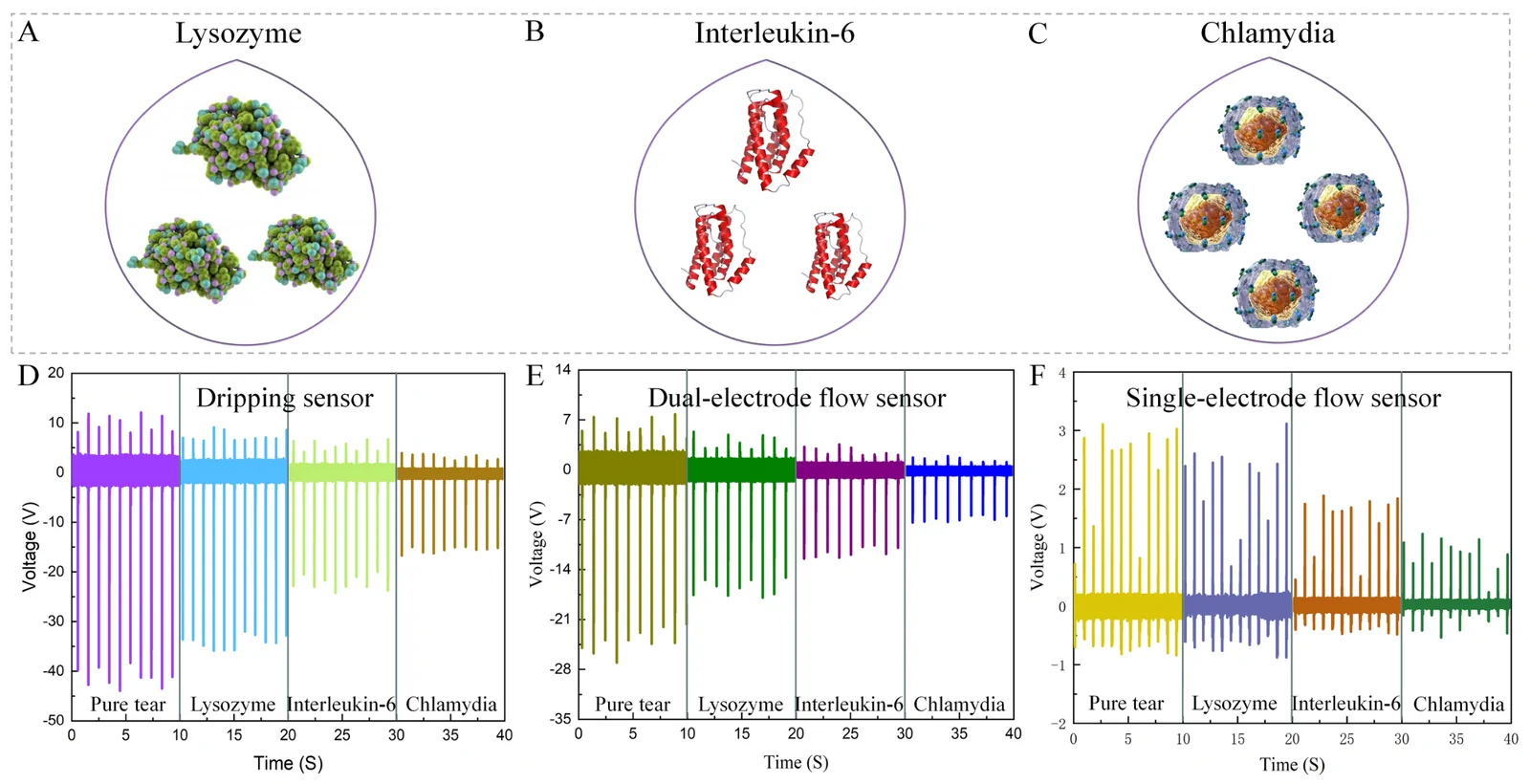
Performance evaluation of multimodal detection of tear biomarkers using the MIBET system. Three representative biomarkers, (**A**) lysozyme (for dry eye disease), (**B**) interleukin-6 (for autoimmune ophthalmopathy), and (**C**) Chlamydia (for trachoma), were individually spiked into an artificial tear matrix for targeted detection using three types of TENG sensors. (**D**–**F**) Comparison of output signals of three types of TENG sensors. Distributions of negative peak voltages from droplet-mode, dual-electrode sliding-mode, and single-electrode sliding-mode TENG sensors across four sample groups, revealing distinct influences of different biomarkers on the triboelectric output characteristics.

To systematically evaluate the clinical application potential of the MIBET system for tear biomarker detection, a series of multimodal targeted sensing experiments was conducted. Three representative biomarkers closely associated with ophthalmic diseases were individually incorporated into an artificial tear matrix: lysozyme (a marker of dry eye disease), interleukin-6 (associated with autoimmune ophthalmopathy), and Chlamydia antigen (corresponding to trachoma), as shown in Figure 6A–C. Variations in the concentrations of these biomarkers can provide important diagnostic evidence for clinical ophthalmology. As shown in Figure 6D–F, the recognition performance of the MIBET system for target analytes was systematically evaluated using multimodal triboelectric sensing. The results indicate that the sensor output signals (negative peak voltage) exhibit distinct and concentration-dependent responses to different biomarkers, with each sensing mode showing unique signal characteristics: (1) For the droplet-based mode, the peak output voltages for four sample groups (pure artificial tears, and lysozyme-, interleukin-6-, and Chlamydia-doped artificial tears) are 40–45 V, 30–35 V, 20–25 V, and 15 V, respectively. Lysozyme doping induces the smallest voltage reduction (~22.5%), whereas Chlamydia causes the most pronounced signal attenuation (~66%), indicating a graded sensitivity of this mode toward different biomarkers. (2) The dual-electrode sliding mode exhibits output voltages of 25–28 V, 16–18 V, 10–12 V, and 7 V, respectively. Notably, Chlamydia induces an approximately 70% voltage decrease, significantly larger than that caused by the other two biomarkers, suggesting higher sensitivity. (3) The single-electrode sliding mode generates relatively low output voltages, typically below 1 V. Despite its low absolute signal amplitude, this mode exhibits the most pronounced concentration-dependent response upon Chlamydia antigen spiking, highlighting its strong potential for detecting low-abundance biomarkers. Comparative analysis of the three sensing modes indicates that different biomarkers exert distinct influences on the triboelectric effect. Positively charged lysozyme primarily modulates the output signal by altering the surface charge density of droplets. Neutral interleukin-6 may influence the signal response by regulating the interfacial adhesion between droplets and electrode materials. In contrast, negatively charged Chlamydia antigen markedly suppresses the efficiency of triboelectric charge transfer. These distinct mode-dependent responses provide a theoretical basis for the selective discrimination and quantitative detection of multiple tear biomarkers.

To further evaluate the quantitative detection capability of the system for different biomarkers, concentration-gradient experiments were conducted using lysozyme, IL-6, and Chlamydia antigen. For each concentration, at least three independent replicates were performed to ensure statistical reliability. Calibration curves for the three biomarkers obtained using the three TENG sensing modes were established, and key analytical parameters, including the limit of detection (LOD), sensitivity, linear range, and reproducibility, were quantified, as summarized in Section S4 of the Supporting Information.

### 3.5. Innovations, Limitations, and Future Work

The primary innovation of this study is the development of a multimodal integrated biomimetic triboelectric electronic tongue for intelligent tear analysis. Based on the liquid–solid triboelectric effect, the platform directly converts interfacial interactions between tear droplets and the PTFE surface into measurable electrical signals. Compared with conventional tear analysis methods, this strategy offers several advantages, including a simple structure, low cost, reagent-free operation, and self-powered sensing capability, thereby providing a promising technical route for portable, home-based, and point-of-care ophthalmic testing. A key feature of the MIBET system is the integration of three complementary TENG modes on a single microfluidic chip: droplet-impact mode, dual-electrode sliding mode, and single-electrode sliding mode. These modes capture the interfacial physicochemical characteristics of tear samples from different perspectives, generating rich and complementary sensing information. This multimodal sensing strategy contributes to improved accuracy, stability, and intelligent recognition capability in tear analysis.

Nevertheless, several limitations remain in this study. First, the current MIBET system is still at the stage of sensor characterization and proof-of-concept validation. In this work, deionized water, artificial tears, and biomarker-spiked artificial tears were used as model samples to investigate the sensing mechanism, optimize device parameters, evaluate signal stability, and establish preliminary machine learning models. Although these samples enable controlled and reproducible performance evaluation, they cannot fully replicate the complex composition, dynamic secretion behavior, inter-individual variability, and matrix effects of real human tears. Therefore, the present results primarily demonstrate the feasibility of the proposed strategy and should not yet be considered direct evidence of clinical diagnostic capability. Second, continuous droplet addition was used to assess signal stability, repeatability, and parameter dependence. However, in practical clinical scenarios, tear samples are typically available only at the microliter scale, which is much lower than the sample volume used for device characterization and optimization. Therefore, the testing procedure should be further optimized to reduce sample consumption and better align with realistic tear collection conditions.

Future work will focus on device optimization, real-sample validation, and intelligent data analysis. First, the microfluidic sampling module and device structure will be further optimized to improve miniaturization and integration while reducing tear sample consumption. After optimizing the device and testing procedure, real human tear samples will be evaluated. To improve signal stability, the PTFE triboelectric interface may be pre-activated with deionized water or standard solutions before introducing tear samples, allowing surface charges to accumulate and the device to reach a stable working state. Subsequently, only one or a few microliter-scale tear droplets will be introduced into the MIBET platform for detection, thereby minimizing sample consumption while maintaining stable output. With ethical approval and informed consent, tear samples will be collected from healthy volunteers and patients with ophthalmic diseases using clinically acceptable methods, such as microcapillary tubes, Schirmer strips, or tear meniscus sampling. The obtained multimodal electrical fingerprints will be compared with standard clinical assays to evaluate the reliability, anti-interference capability, and diagnostic relevance of the MIBET platform. Finally, AI-based data analysis will be further improved by establishing a larger and more clinically representative tear database and incorporating systematic feature extraction, algorithm comparison, cross-validation, and external validation to enhance model robustness, generalization, and clinical applicability.

## 4. Conclusions

This work presents a biomimetic triboelectric electronic tongue constructed on a microfluidic chip, integrating three TENG sensing modes, including dripping, dual-electrode sliding, and single-electrode sliding, for the detection of tear pH and disease-related biomarkers. Key operational parameters (drop height: 5–14 cm, tilt angle: 15–60°, drop rate: 45–105 drops/min) were optimized to restrict signal fluctuation of all modes within ±10%. The dripping mode achieved a peak voltage exceeding 60 V at a drop height of 14 cm. The dual-electrode sliding mode exhibited a 266.67% increase in output voltage as the tilt angle decreased from 60° to 15°, while the single-electrode sliding mode showed a monotonic increase in signal output with increasing droplet frequency. Coupled with a random forest algorithm, the three sensing modes achieved pH classification accuracies of 96.6%, 97.8%, and 99.4%, respectively. For the quantification of lysozyme, IL-6, and Chlamydia antigen, the negative voltage peak was used as the readout signal. Among the three sensing modes, Section S1 exhibited the best reproducibility, full-range linearity (R^2^ > 0.99), and the highest overall sensitivity, as well as the lowest LOD for Chlamydia antigen detection, reaching 4250 copies/mL. Section S2 showed acceptable repeatability, with all RSD values below 10%, full-range linear responses, and the lowest LODs for lysozyme and IL-6 detection. In contrast, Section S3 showed relatively poor repeatability at low and medium concentrations. Overall, the MIBET system demonstrates excellent stability, multimodal discrimination capability, and quantitative sensing performance, with Section S1 identified as the optimal channel for universal quantitative biomarker detection. This compact and intelligent platform shows strong potential for tear-based biomarker analysis and ophthalmic disease screening.

## Data Availability

The data presented in this study are available on request from the corresponding author.

## Supplementary Materials

The following supporting information can be downloaded at: https://www.mdpi.com/article/10.3390/bios16080402/s1. Section S1: The exact number of samples for each pH group; Section S2: Use both 5-fold and 10-fold cross-validation to re-evaluate the model performance; Section S3: Comparative analysis of algorithmic performance with previously reported studies; Section S4: Calibration curves and key analytical parameters for biomarker detection.
