# Supplementary material for: Multi-Mode Integrated Bioinspired Electronic Tongue for Point-of-Care Tear Diagnosis
Source: Biosensors (Basel). 2026 Jul 24;16(8):402. doi: 10.3390/bios16080402 (PMC13510568; doi:10.3390/bios16080402)
Supplement: Supplementary file 1 [file biosensors-16-00402-s001.zip › biosensors-4408063-supplementary.pdf]

Multi-Mode Integrated Bioinspired Electronic Tongue for Point-of-Care Tear Diagnosis

S1. The exact number of samples for each pH group.

Table S1. Exact sample numbers for each pH group.

Sensor mode	pH value	Total number of samples	Training set samples	Test set samples
Dripping sensor	6.8	157	110	47
	7.2	182	127	55
	7.6	175	123	52
	8.0	168	118	50
Dual-electrode sliding sensor	6.8	142	99	43
	7.2	158	111	47
	7.6	151	106	45
	8.0	147	103	44
Single-electrode sliding sensor	6.8	125	88	37
	7.2	158	111	47
	7.6	134	94	40
	8.0	130	91	39

It should be noted that stratified sampling was applied during dataset splitting to maintain consistent pH class ratios between the training and test subsets, thereby minimizing evaluation bias caused by uneven class distribution.

S2 Use both 5-fold and 10-fold cross-validation to re-evaluate the model performance

Compared with a single 7:3 hold-out data split, cross-validation reduces the influence of random data partitioning and provides a more objective assessment of model generalization. Specifically, all samples were stratified by pH label to ensure consistent class proportions for pH 6.8, 7.2, 7.6, and 8.0 in each fold. In the 5-fold cross-validation, four folds were used for training, and one fold was used for testing, and this process was repeated five times. In the 10-fold cross-validation, nine folds were used for training, and one fold was used for testing, and the process was repeated ten times. The results showed that the model achieved high average accuracies of 96.5% and 96.6% in the 5-fold and 10-fold cross-validation, respectively. Moreover, stable F1-scores were obtained across all pH classes, confirming the robust generalization capability of the model.

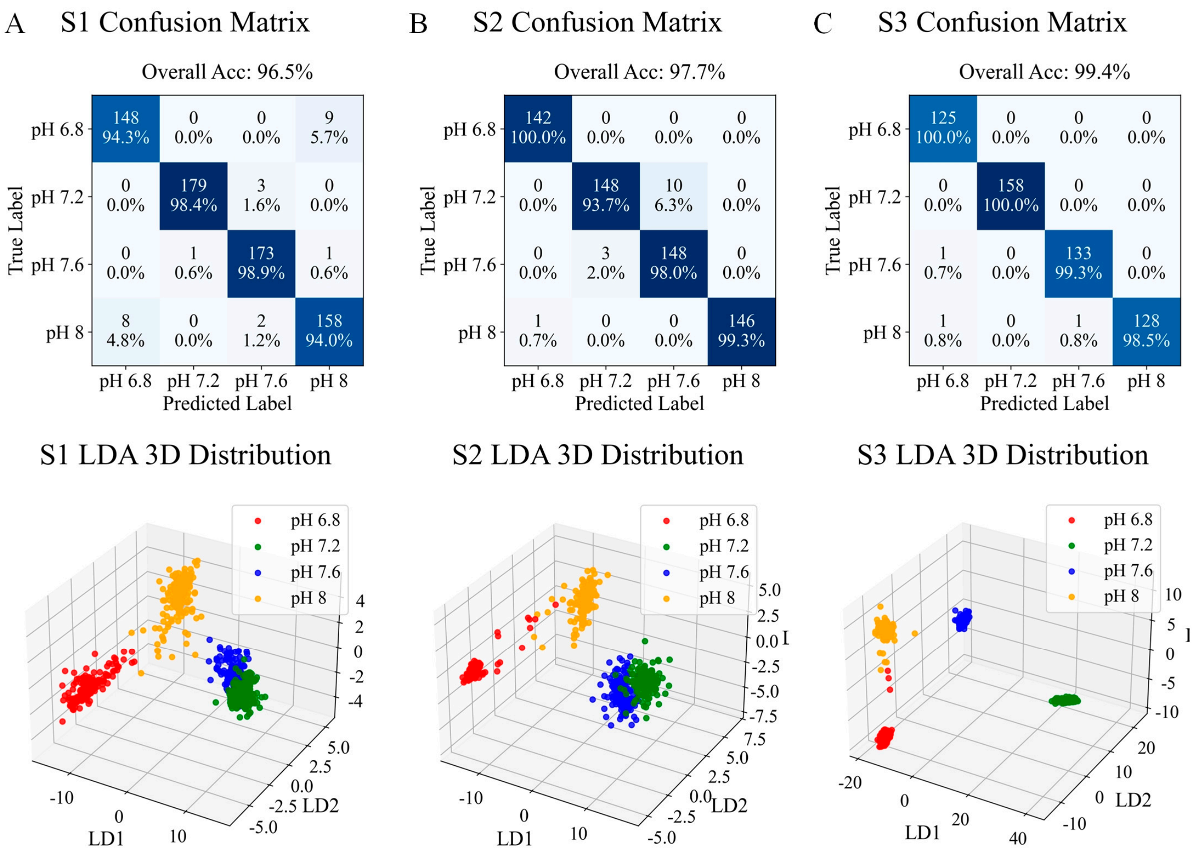

Figure S1. Classification results and feature dimensionality reduction obtained using 5-fold cross-validation for the three TENG sensors. Confusion matrices and LDA visualizations are presented for (A) the droplet-based mode, (B) the dual-electrode sliding mode, and (C) the single-electrode sliding mode.

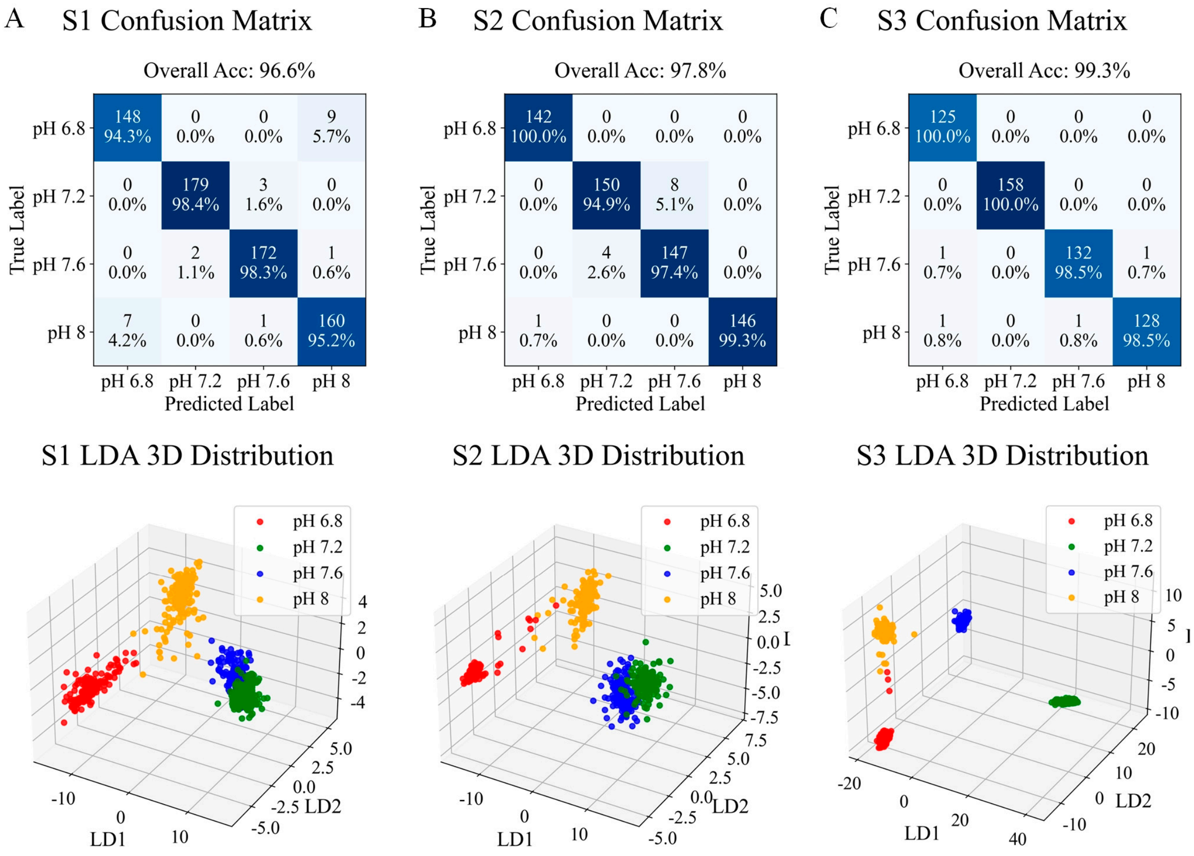

Figure S2. Classification results and feature dimensionality reduction obtained using 10-fold cross-validation for the three TENG sensors. Confusion matrices and LDA visualizations are presented for (A) the droplet-based mode, (B) the dual-electrode sliding mode, and (C) the single-electrode sliding mode.

S3 Comparative analysis of algorithmic performance with previously reported studies

Previous studies have demonstrated the feasibility of combining triboelectric or colorimetric sensing with machine learning for liquid identification and tear analysis. Liu et al. reported a bioinspired triboelectric electronic tongue based on liquid–solid triboelectric signals and machine learning, achieving classification accuracies of 100%, 98.2%, 97.0%, and 96.9% for chemical solutions, environmental samples, tea samples, and NaCl solutions with different concentrations, respectively [20]. The work confirmed the potential of triboelectric electronic tongues for intelligent liquid identification. In comparison, our study extends multimodal TENG sensing to artificial tear pH analysis and ophthalmic biomarker-related model samples. The three TENG sensing modes achieved pH classification accuracies of 96.6%–99.4%, indicating that multimodal triboelectric signals can serve as effective electrical fingerprints for artificial tear pH recognition. For AI-assisted tear analysis, Wang et al. developed a microfluidic colorimetric wearable sensor for the simultaneous

detection of vitamin C, H^+/pH, Ca^{2+}, and proteins in tears [16]. Their CNN-GRU model corrected errors caused by color temperature and pH variations, enabling quantitative prediction using approximately 20 μL of tear samples. Although this approach enables multi-analyte detection in real tear samples, it relies on colorimetric reactions, optical readout, and image correction. In contrast, our method uses self-powered triboelectric signals as the sensing output, avoiding colorimetric reagents and complex optical systems, which may benefit portable, low-power, and reagent-free tear analysis. Xiong et al. reported a machine-learning-assisted pH detection method based on pH test strips [14]. By extracting image features from test strips, their method improved pH-reading reliability under variations in illumination, image acquisition conditions, and subjective interpretation. In contrast, our study does not rely on color changes or image acquisition. Instead, pH classification is achieved using dynamic voltage waveforms generated during droplet–PTFE contact electrification and sliding. These signals are influenced by pH-dependent charge transfer, conductivity, ionic strength, electric double-layer modulation, and droplet motion behavior, thus reflecting the multidimensional interfacial electrical characteristics of artificial tear samples.

Table S2. Comparison of algorithmic results between this work and previously reported studies.

Reference/ this work	Method/system	Samples/targets	Signal or features	Algorithms used	Reported algorithm results	Main advantages
[20]	Bioinspired integrated triboelectric electronic tongue based on liquid-solid triboelectric sensing	Chemical solutions, environmental samples, tea samples, and NaCl solutions with different concentrations	Triboelectric electrical signals generated from multiple triboelectric sensing channels	LDA, SVC/SVM, and Random Forest	Chemical solutions: 100%; environmental samples: 98.2%-98.3%; tea samples: 97.0%; NaCl concentration classification: 96.9%	High-accuracy liquid identification using self-powered triboelectric fingerprints and machine learning
[16]	AI-assisted microfluidic colorimetric wearable sensor system for tear analysis	Human tear biomarkers, including vitamin C, H^+/pH, Ca^{2+}, and proteins	Colorimetric image / RGB signals acquired by smartphone imaging	CNN-GRU deep learning model	Test-set R^2 reached 0.998 for pH prediction and 0.994 for the other three biomarkers; approximately 20 μL tear samples were used	Quantitative tear biomarker analysis using AI-assisted colorimetric imaging

[14]	Machine-learning-assisted pH detection based on commercial pH test strips	pH solutions measured by pH indicator strips	Image/color features extracted from pH test strip photographs	Machine-learning models for quantitative pH determination	Improved the accuracy and reliability of pH determination using pH strips under image-acquisition variations	Low-cost pH detection with improved reading reliability
This work	Multi-mode integrated bioinspired triboelectric electronic tongue on a microfluidic chip	Artificial tears with different pH values; artificial tears doped with lysozyme, IL-6, and Chlamydia antigen	Dynamic voltage waveforms from three TENG modes: droplet-based mode, dual-electrode sliding mode, and single-electrode sliding mode	LDA for dimensionality reduction and visualization; Random Forest for classification	Artificial tear pH classification accuracies of 96.6%, 97.8%, and 99.4% for the three TENG modes, respectively	Reagent-free multimodal triboelectric sensing with complementary electrical fingerprints

S4. Calibration curves and key analytical parameters for biomarker detection

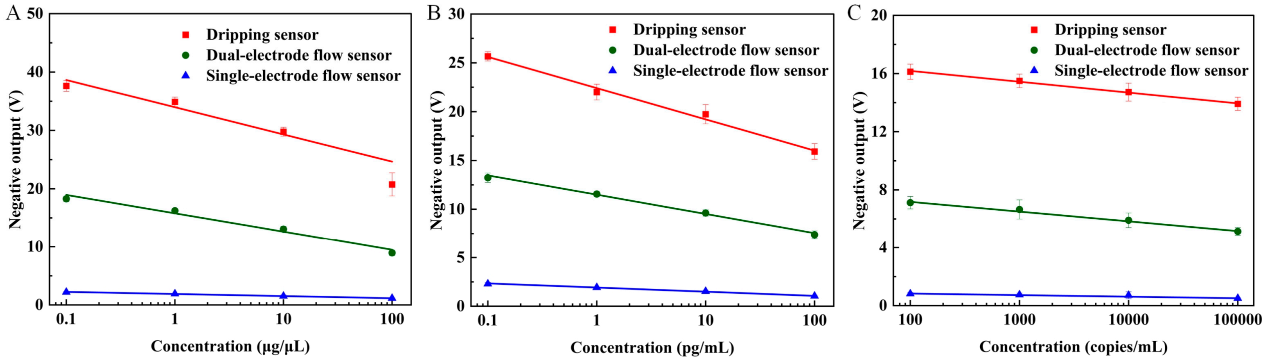

Figure S3. Calibration curves for three biomarkers measured using the three TENG sensors: (A) lysozyme, (B) interleukin-6, and (C) Chlamydia antigen. Data are presented from at least three repeated measurements (n ≥ 3).

The calculation formulas for each parameter are as follows:

Reproducibility (RSD):
$$RSD = \frac{SD}{Mean} \times 100\% \qquad \text{(S1)}$$

Linear Range
$$R^2 \geq 0.99 \qquad \text{(S2)}$$

Sensitivity（S）
$$S = \frac{\Delta v}{\Delta c} \qquad \text{(S3)}$$

Limit of detection (LOD)
$$LOD = \frac{3\sigma}{S} \qquad \text{(S4)}$$

The calculated results are as follows:

Table S3. Evaluation of reproducibility using the relative standard deviation (RSD) calculated from three parallel replicates at each concentration.

Lysozyme				IL-6				Chlamydia antigen			
TENG sensors	S1	S2	S3	TENG sensors	S1	S2	S3	TENG sensors	S1	S2	S3
0.1 μg/mL	2.47	2.62	4.77	0.1 pg/mL	1.85	3.59	6.24	10^2 copies/mL	3.25	6.02	11.06
1 μg/mL	2.34	2.25	3.62	1 pg/mL	3.66	2.11	3.14	10^3 copies/mL	3.01	9.97	14.97
10 μg/mL	2.53	3.45	13.59	10 pg/mL	5.01	3.32	7.30	10^4 copies/mL	4.20	8.57	33.00
100 μg/mL	9.56	5.17	5.71	100 pg/mL	5.04	5.46	8.14	10^5 copies/mL	3.24	5.15	7.49

Note: RSD values greater than 10% indicate substantial variation among parallel replicates, suggesting insufficient repeatability. S1–S3 represent the three TENG sensors. The relative standard deviation (RSD) was calculated as SD/mean × 100% and expressed as a percentage (%).

Table S4. Evaluation of the linear range using three parallel replicates at each concentration.

Lysozyme			IL-6			Chlamydia antigen		
S1	S2	S3	S1	S2	S3	S1	S2	S3
0.1–100 μg/mL	0.1–100 μg/mL	0.1–100 μg/mL	0.1–100 pg/mL	0.1–100 pg/mL	0.1–100 pg/mL	10^2–10^5 copies/mL	10^2–10^5 copies/mL	/

Note: S1–S3 represent the three TENG sensors.

Table S5. Evaluation of sensitivity using three parallel replicates at each concentration.

Samples	S1	S2	S3
Lysozyme	0.1690 V·mL/μg	0.0932 V·mL/μg	0.0106 V·mL/μg
IL-6	0.096 V·mL/pg	0.0514 V·mL/pg	0.0123 V·mL/pg
Chlamydia antigen	2.40×10^{-5} V·mL/copies	1.97×10^{-5} V·mL/copies	3.02×10^{-6} V·mL/copies

Note: S1–S3 represent the three TENG sensors.

Table S6. Evaluation of the limit of detection (LOD) using three parallel replicates at each concentration.

Samples	S1	S2	S3
Lysozyme	0.0087 μg/mL	0.0028 μg/mL	0.0119 μg/mL
IL-6	0.0052 pg/mL	0.0031 pg/mL	0.0095 pg/mL
Chlamydia antigen	4250 copies/mL	5787 copies/mL	/

The quantitative performance of the three sensing modes, namely S1, S2, and S3, was evaluated using lysozyme, IL-6, and Chlamydia antigen as representative analytes. The averaged maximum and minimum output voltages were used as response signals. Among the three sensing channels, S1 exhibited the best reproducibility, with RSD values below 5.01% for all three analytes at low and medium concentrations. This was followed by S2, which showed RSD values below 10%. In contrast, S3 exhibited poor repeatability at low and medium concentrations, with RSD values exceeding 10%. Both S1 and S2 achieved full-range linear responses for all target analytes, with R^2 values greater than 0.99, whereas S3 failed to meet the linearity criterion for Chlamydia antigen detection. In terms of sensitivity, the overall performance followed the order of S1 > S2 > S3 for all analytes. S2 provided lower LODs for lysozyme and IL-6, while S1 achieved the optimal LOD for Chlamydia antigen detection, reaching 4250 copies/mL. Overall, this comprehensive comparison indicates that S1 is the preferred sensing channel for universal quantitative detection of the three biomolecules, whereas S3 is only suitable for qualitative detection of high-concentration samples.
